# Patterns of kidney supportive care referrals in a tertiary hospital in India: a six-year audit

**DOI:** 10.1186/s12882-026-05066-x

**Published:** 2026-05-25

**Authors:** S. Gayatri, Arun Ghoshal, Anuja Damani, Shreya Nair, Shailja Sharma, Trishtha Agarwal, Aahaan Agarwal, Krithika S. Rao, N. Shankar Prasad, Naveen Salins

**Affiliations:** 1 Department of Palliative Medicine and Supportive Care, Kasturba Medical College, Manipal Academy of Higher Education, Manipal, 576104 India; 2 Kasturba Medical College, Manipal Academy of Higher Education, Manipal, 576104 India; 3 Department of Nephrology, Kasturba Medical College, Manipal Academy of Higher Education, Manipal, 576104 India

**Keywords:** Advance care planning, End-stage kidney disease, Goals-of-care, Palliative integration, Kidney supportive care, India

## Abstract

**Background:**

Patients with end-stage kidney disease (ESKD) and acute kidney injury on chronic kidney disease (AKI-on-CKD) experience significant symptom burden and high mortality, yet evidence for structured palliative integration within nephrology remains limited in low- and middle-income countries.

**Aim:**

To describe the timing and reasons for referrals from Nephrology to Palliative Medicine, the interventions delivered, and clinical outcomes following referral.

**Methods:**

A retrospective cohort study was conducted of all adult patients (*n* = 325) referred from Nephrology to Palliative Medicine. Data included demographics, renal diagnosis, dialysis modality, comorbidities, treatment intent, and documented palliative interventions, and were analyzed using descriptive statistics.

**Results:**

Mean age was 63.6 ± 14.1 years; 229 (70.5%) were male. Renal diagnoses comprised ESKD (215; 66.2%), AKI-on-CKD (86; 26.5%), and AKI (16; 4.9%). Dialysis modalities included maintenance hemodialysis (194, 59.7%), dialysis for AKI (63, 19.4%), and conservative kidney management (21, 6.5%). Referrals were primarily for GOC discussions (41.5%), followed by pain and symptom management (29.2%) and end-of-life (EOL) care (27.4%). Referrals specifically for conservative kidney management were uncommon (1.8%). Among deaths, 154 (75%) occurred in the hospital. Common triggers for referral included sepsis, multiorgan failure, and decisions to stop dialysis.

**Conclusion:**

Palliative care involvement predominantly occurred during acute decline—often related to sepsis, multiorgan failure, or dialysis withdrawal. Although most patients died in the hospital, the high frequency of GOC and EOL discussions highlights the need for earlier, proactive palliative care engagement to support informed decision-making, symptom relief, and care aligned with patient preferences.

## Introduction

CKD is a long-term, progressive condition characterized by gradual functional decline, punctuated by intermittent episodes of acute deterioration—often leading to hospitalization and an inability of the patient to regain their previous level of functioning [1, 2]. Globally, CKD affected an estimated 788 million people aged 20 years and older in 2023, up from 378 million in 1990 [3]. The prevalence of CKD in India is estimated to be around 13.24% [4]. As kidney function declines, patients experience a substantial symptom burden—including pain, pruritus, fatigue, sleep disturbances, nausea, dyspnea, and psychological distress—that often rivals or exceeds that seen in advanced cancer [5]. Individuals with End Stage Kidney Disease (ESKD) undergoing dialysis often face multiple distressing symptoms related to the underlying condition, dialysis therapy, and coexisting illnesses [5–7]. This cumulative burden results in reduced quality of life and is correlated with increased future hospitalization and mortality, underscoring the value of timely supportive care interventions [5, 8].

Multiple models of Kidney Supportive Care (KSC) integration have been described in the literature, including multidisciplinary care structures, formal palliative care consultation services, and standardized supportive care pathways [9]. Effective implementation of KSC requires targeted education and capacity-building among healthcare providers, alongside the use of validated tools for symptom assessment and management [10–13]. Embedding advance care planning (ACP) within routine clinical encounters, coupled with structured communication training, has been shown to strengthen goal-concordant care and improve the quality of decision-making for patients and families [9, 14].

Despite high needs, palliative care remains underutilized in CKD when compared to oncology and other chronic illnesses [11, 15]. Prior studies have also demonstrated low rates of nephrology referral to palliative care services [2, 15]. A qualitative meta-synthesis of healthcare professionals’ perspectives and attitudes identified several challenges in implementing KSC, including professional role challenges, difficulties navigating complex clinical decisions, communication concerns, organizational factors, and the imperative to provide patient-centered, ethically appropriate care [16].

Timely Palliative Care (PC) involvement can improve symptom control, facilitate advance care planning, reduce unnecessary hospitalizations, and align treatment with patient values and goals—especially around decisions such as continuing or withdrawing dialysis and end- of- life transitions [14, 15, 17–19]. 

Palliative care integration in nephrology continues to be an emerging area of practice, predominantly documented in high-income countries such as the USA, while evidence from low-income countries is notably absent [20]. In India, KSC is delivered through both integrative and consultative models, although structured integration within nephrology remains limited and varies across regions and institutions. Services commonly include holistic symptom management, psychosocial and spiritual support, discussions about goals of care, and advance care planning. At our institution, PC services are completely external to the nephrology department, and referrals are made to the Department of Palliative Medicine on a consultative basis. Referrals are initiated by nephrology consultants based on clinical triggers identified during patient assessment, such as high symptom burden, dialysis-related complications, poor prognosis, or the need for goals-of-care discussions and end-of-life planning. This study aims to describe the reasons for referrals from Nephrology to Palliative Medicine, to evaluate the palliative care interventions provided, and the clinical outcomes following referral.

## Objectives

1. **Primary objective**
  To describe the reasons for referrals from the Department of Nephrology to the Department of Palliative Medicine.

2. **Secondary objective**
  - To evaluate the palliative care interventions delivered following PC referral.
  - To assess treatment-related and dialysis-related outcomes following PC referral.
  - To assess the clinical outcomes following PC referral.

## Methods

### Study design and setting

This retrospective, observational study was conducted at a tertiary academic teaching hospital in southern India, encompassing all adult patients referred from the Department of Nephrology to the Department of Palliative Medicine and Supportive Care between January 2019 and December 2024. The institution houses a multidisciplinary palliative medicine team, integrated with inpatient and outpatient services, that provides consultation across various clinical specialties. Supportive-care services include comprehensive symptom assessment and management, psychosocial support, advance-care planning (ACP), and goals-of-care (GOC) discussions.

### Participants

All patients aged 18 years or older who received a referral from Nephrology to Palliative Medicine during the study period. Referral sources included inpatient wards, ICU, HDU, and the emergency department. Exclusion criteria were: (i) admissions under Nephrology without a documented referral to Palliative Medicine, and (ii) incomplete or missing documentation of the referral process or clinical course.

### Data collection

Data were extracted from the institutional electronic medical record and entered into a structured spreadsheet. Collected variables included demographic characteristics (age, sex), primary diagnosis, disease category (like CKD, ESKD, etc.), comorbidities, documented reason(s) for referral, palliative care interventions delivered, and outcome at discharge (home/OPD follow-up, transfer to hospice/local hospital, death, or discharge against medical advice).

### Outcome measures

The primary outcome of the study was the distribution of documented reasons for referral from the Department of Nephrology to the Department of Palliative Medicine, as recorded in electronic medical records. Referral reasons were categorized into: (i) goals-of-care (GOC) discussions, (ii) pain and symptom management, (iii) end-of-life (EOL) care, and (iv) conservative kidney management (CKM).

### Secondary outcomes included

Palliative care interventions delivered, categorized as GOC discussions, pain and symptom management, end-of-life care, and advance care planning (ACP). Interventions were identified based on documentation in clinical notes during the palliative care consultation.

Dialysis-related outcomes following palliative care referral, including continuation of dialysis, withholding (non-initiation) of dialysis, withdrawal of dialysis, initiation of dialysis, and transition to conservative kidney management. These outcomes were assessed separately for clinically relevant subgroups: end-stage kidney disease (ESKD), acute kidney injury (AKI), and AKI in patients with chronic kidney disease (AKI-on-CKD).

Clinical outcomes following palliative care referral are defined as: (i) continuation of active treatment, (ii) withdrawal of life-sustaining treatment (including dialysis and other organ support where documented), (iii) transition to conservative kidney management, (iv) symptom improvement, and (v) discharge with supportive care (home or local hospital).

Mortality-related outcomes, including in-hospital mortality and place of death (hospital vs. home), were documented.

All outcomes were derived from routinely documented electronic medical records. Where multiple referral reasons or interventions were documented, all relevant categories were captured.

### Statistical analysis

Data were analyzed using Python (version 3.11; Python Software Foundation, Wilmington, DE, USA) with pandas (v2.0), NumPy (v1.25), and scikit-learn (v1.3). Descriptive statistics were used to summarize baseline characteristics and outcomes. Categorical variables (including sex, diagnosis category, referral source, dialysis status, referral reasons, interventions, and clinical outcomes) were reported as frequencies and percentages. Age was summarized using mean and standard deviation (SD).

Given the clinical heterogeneity of the cohort, subgroup analyses were performed for patients with ESKD, AKI, and AKI-on-CKD to examine differences in referral patterns, interventions, dialysis-related decisions, and outcomes across these groups.

Associations between dialysis modality and dialysis-related outcomes were assessed using the Fisher–Freeman–Halton exact test, given small subgroup sizes and categorical data structure.

All statistical tests were two-sided, and p-values < 0.05 were considered statistically significant.

### Ethical considerations

The study was approved by the Institutional Ethics Committee of Kasturba Medical College, Manipal Academy of Higher Education (IEC 226/2024). Given its retrospective design and use of de-identified data, the requirement for individual informed consent was waived. The study adhered to the principles outlined in the Declaration of Helsinki (2013) and local data protection guidelines.

## Results

### Cohort characteristics

A total of 325 adult patients referred from the Department of Nephrology to the Department of Palliative Medicine between January 2019 and December 2024 were included in the analysis (Table 1). All referrals analyzed were from inpatient settings, including wards, intensive care units (ICU), high-dependency units (HDU), and the emergency department.

**Table 1:** Baseline demographic and diagnostic characteristics of the cohort (*N* = 325)

Characteristic	Category	*n* (%)
Age (years)	—	63.6 ± 14.1 (Mean ± SD)
Sex	Male	229 (70.5%)
	Female	96 (29.5%)
	Male: Female	≈ 2.39: 1
Insurance status	Full insurance	80 (24.6%)
	Partial insurance	103 (31.7%)
	No insurance (out-of-pocket)	142 (43.7%)
Renal diagnosis	End-stage kidney disease (ESKD)	215 (66.2%)
	AKI on CKD	86 (26.5%)
	AKI	16 (4.9%)
	Others	8 (2.4%)
Current diagnosis (reason for admission)	Sepsis / infection-related	176 (54.2%)
	Cardiovascular event	42 (12.9%)
	Metabolic/uremic complications	58 (17.8%)
	Neurological (encephalopathy/stroke)	16 (4.9%)
	Others	33 (10.2%)
Dialysis status	Maintenance haemodialysis (MHD)	194 (59.7%)
	Peritoneal dialysis (PD)	6 (1.8%)
	AKI on dialysis	63 (19.4%)
	AKI not on dialysis	41 (12.6%)
	Conservative kidney management (CKM)	21 (6.5%)
Charlson Comorbidity Index (CCI)	0 (no comorbidities)	3 (1%)
	1–2 (mild burden)	14 (4.3%)
	3–4 (moderate burden)	64 (19.7%)
	≥ 5 (severe burden)	244 (75%)
Patient Location	Ward	165 (50.8%)
	ICU	131 (40.3%)
	HDU	27 (8.3%)
	Emergency	2 (0.6%)
Year of referral	2019	31 (9.5%)
	2020	26 (8%)
	2021	53 (16.3%)
	2022	56 (17.2%)
	2023	62 (19%)
	2024	97 (29.9%)

The cohort’s mean age was 63.6 years, and 229 patients (70.5%) were male. A substantial proportion of patients lacked comprehensive financial coverage: 142 (43.7%) paid entirely out of pocket, while only 80 (24.6%) had full insurance coverage that adequately covered dialysis and hospitalization expenses with minimal or no out-of-pocket costs. Patients with partial insurance had dialysis costs partly or fully reimbursed depending on the scheme, but continued to incur out-of-pocket expenses for medications, investigations, procedures, consumables, and hospitalization.

End-stage kidney disease (ESKD) was the most common diagnosis (215, 66.2%), followed by AKI on CKD (86, 26.5%) and AKI (16, 4.9%). The cohort had a high comorbidity burden, with 244 patients (75%) having a Charlson Comorbidity Index (CCI) ≥ 5.

Most patients (194, 59.7%) were receiving maintenance hemodialysis at the time of referral, with a mean dialysis duration of 3.4 years, while 21 patients (6.5%) were managed with conservative kidney management (CKM). Referrals originated primarily from inpatient wards (50.8%) and ICUs (40.3%). The number of referrals increased over time, from 31 in 2019 to 97 in 2024.

### Primary outcome: Reasons for referral to palliative medicine

The most common documented reason for referral was goals-of-care (GOC) discussions (135, 41.5%), followed by pain and symptom management (95, 29.2%) and end-of-life (EOL) care (89, 27.4%). Referrals specifically for conservative kidney management (CKM) were uncommon (6, 1.8%) (Table 2).

**Table 2:** Reasons for palliative medicine referral

Reason for Referral	*n* (%)
Goals-of-care (GOC) discussions	135 (41.5%)
Pain/symptom management	95 (29.2%)
End-of-life (EOL) care	89 (27.4%)
Conservative kidney management	6 (1.8%)

### Subgroup analysis by disease category

#### Referral patterns varied across clinical subgroups

Among patients with AKI, the most common reasons for referral were EOL care (50%), GOC discussions (25%), and symptom management (25%).

In AKI on CKD, GOC discussions were the predominant indication (55.8%), followed by symptom management (23.3%) and EOL care (20.9%).

In ESKD, GOC discussions remained the leading reason (36.9%), followed by symptom management (32.7%), EOL care (27.6%), and CKM (2.8%).

#### Secondary outcome 1: Palliative care interventions delivered

**Fig. 1:**
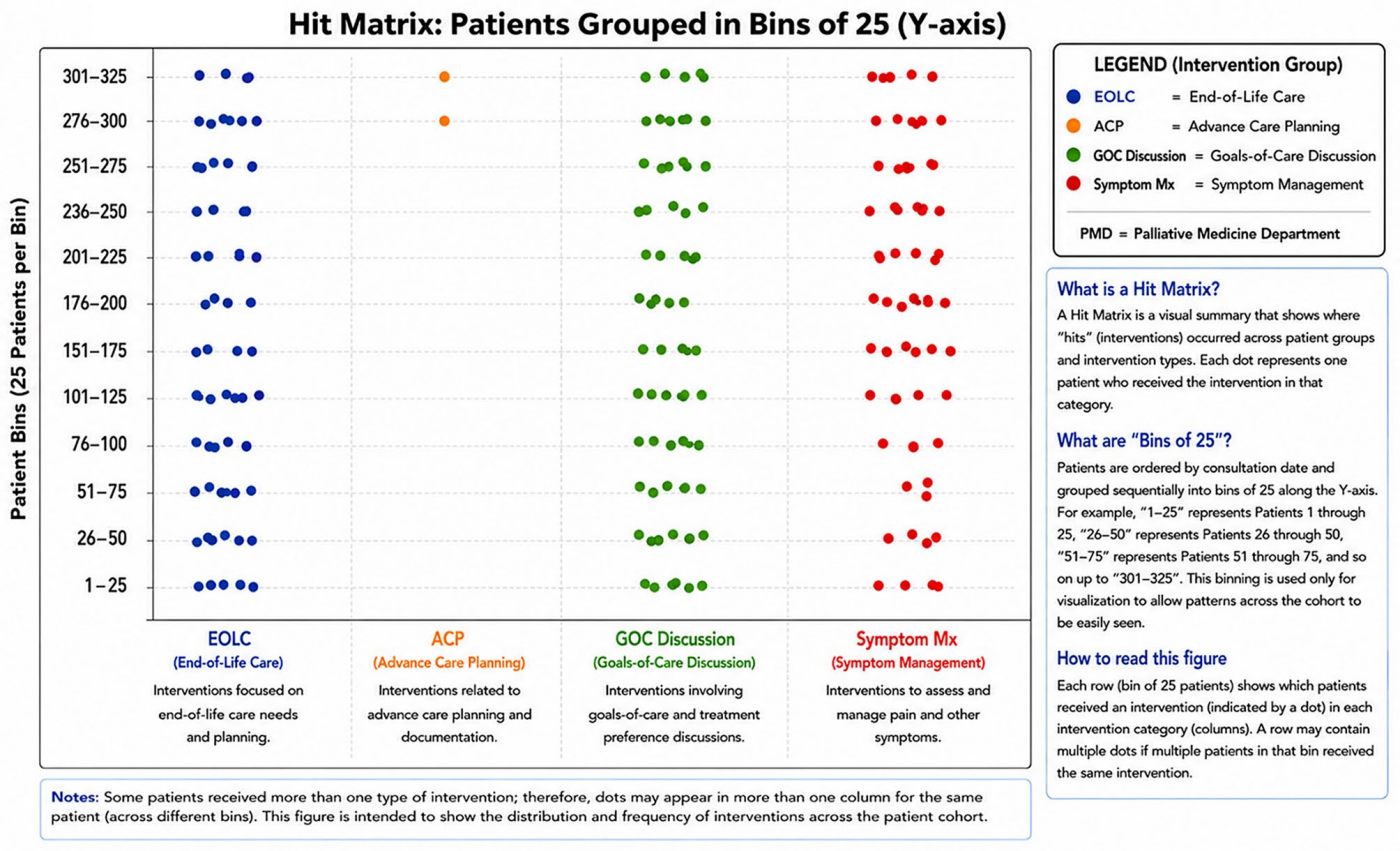
Patient-wise Hit-matrix of PMD interventions

The most frequently documented intervention was GOC discussions (254, 78.2%), followed by end-of-life care (163, 50.2%) and pain and symptom management (145, 44.6%). Advance care planning (ACP) was documented in 2 patients (0.6%) (Fig. 1; Table 3).

**Table 3:** Interventions provided by the PC team

Intervention	*n*
Goals-of-care discussions	254 (78.2)
Pain and symptom control	145 (44.6)
End-of-life care	163 (50.2)
Advance care planning	2 (0.6)

More than one intervention was performed during a single consultation; therefore, percentages may exceed 100%. The average number of interventions per consultation was 1.74

#### Secondary outcome 2: Dialysis-related outcomes following palliative care referral

##### AKI subgroup

Among patients with AKI, 10 (62.5%) did not require dialysis. A statistically significant association was observed between dialysis category and dialysis-related outcomes (Fisher–Freeman–Halton exact test, *p* = 0.018) (Table 4). Patients with AKI not requiring dialysis were more frequently managed conservatively, whereas patients already receiving dialysis were more likely to continue dialysis therapy.

**Table 4:** Correlation between dialysis type and dialysis- related outcomes in patients with AKI

Dialysis Type	Continuation of Dialysis	Withholding Dialysis	Withdrawing Dialysis	Not Indicated	Total
ND-AKI	1	7	0	2	10
D-AKI	4	1	1	0	6
**Total**	5	8	1	2	16

ND-AKI: Non- dialysis dependent AKI D-AKI: Dialysis dependent AKI

##### ESKD subgroup

Among patients with ESKD, the majority were receiving maintenance hemodialysis (180, 83.7%). Following palliative care referral, dialysis-related decisions were primarily continuation of dialysis (93, 43.3%) and withdrawal of dialysis (91, 42.3%). Smaller proportions transitioned to CKM (24, 11.2%) or had dialysis initiated (7, 3.3%) (Table 5).

##### AKI on CKD subgroup

Among patients with AKI on CKD, most had dialysis-requiring AKI (51, 59.3%). The most common dialysis-related outcomes were continuation of dialysis (33, 38.4%) and withdrawal of dialysis (29, 33.7%), followed by CKM (21, 24.4%) and initiation of dialysis (3, 3.5%).

**Table 5:** Frequency of dialysis type in patients with ESKD and dialysis-related outcomes after PC referral

Dialysis type at referral	*n* = 215 (%)	
Maintenance Haemodialysis (MHD)	180 (83.7)	
Conservative Kidney Management (CKM)	20 (9.3)	
Initiation of Haemodialysis (HD)	9 (4.2)	
Peritoneal Dialysis (PD)	6 (2.8)	
Dialysis-related outcome after PC referral	*n* = 215(%)	
Continuation of dialysis	93 (43.3)	
Withdrawal of dialysis	91 (42.3)	
Conservative Kidney Management (CKM)	24 (11.2)	
Initiation of dialysis	7 (3.3)	

#### Secondary outcome 3: Clinical outcomes following palliative care referral

Clinical outcomes following palliative care referral are summarized in Table 6.

**Table 6:** Clinical outcomes following PC referral

Outcome following PC referral	AKI on CKD *n* (%)	ESKD *n* (%)	AKI *n* (%)
Withdrawing LST, including HD	27 (31.4)	51 (23.7)	5 (31.3)
Conservative Kidney Management	18 (20.9)	47 (21.9)	2 (12.5)
Continuation of active treatment	14 (16.3)	20 (9.3)	4 (25)
Symptom improvement	18 (20.9)	78 (36.3)	4 (25)
Continue supportive care at home	7 (8.1)	19 (8.8)	1 (6.3)
Continue supportive care at the local hospital	2 (2.3)		
**Total**	**86 (100)**	**215 (100)**	**16 (100)**

In ESKD, the most frequent outcomes were symptom improvement (78; 36.3%) and CKM foregoing hemodialysis (47; 21.9%).

In AKI on CKD, withdrawal of life-sustaining treatment was most common (27; 31.4%), followed by CKM (18; 20.9%) and symptom improvement (18; 20.9%). In the AKI group, withdrawal of LST, including hemodialysis was the most common outcome (31.3%).

#### Secondary outcome 4: Mortality and place of death

Among the 204 patients with documented place of death, 154 (75.5%) died in hospital, while 50 (24.5%) died at home. Among deceased patients, in-hospital death was more common in insured patients compared to uninsured patients (70.5% vs. 60.4%), whereas home deaths were relatively more frequent among uninsured patients.

## Discussion

The rise in referrals from 2019 to 2024 may indicate greater recognition of KSC’s role in tertiary care nephrology; however, it may also reflect factors such as increasing illness severity, improved insurance coverage and healthcare access, greater availability of PC services, and evolving referral practices. This aligns with global trends emphasizing early palliative care integration as an essential component of comprehensive care for patients with ESKD and multimorbidity [21].

A notable observation in this cohort was that GOC discussions were the primary driver of referrals, underscoring the crucial role of PC services in navigating complex decision-making, particularly regarding the appropriateness of ongoing dialysis, transitions to comfort-focused care, and end-of-life planning. Previous literature has similarly shown that GOC conversations occupy a central place in kidney supportive care, as patients with ESKD often face prognostic uncertainty, high rates of acute deterioration, and a mismatch between aggressive treatment pathways and patient priorities [9]. 

The predominance of communication-based interventions, including GOC discussions, over relatively fewer documented ACP conversations highlights a persistent challenge in nephrology: ACP is often deferred until late in the disease course. This has been widely documented in international studies, where only a minority of patients on dialysis have early ACP discussions despite strong guideline recommendations [22]. Our findings reinforce the need for systematic ACP integration earlier in the disease trajectory.

Clinical outcomes demonstrate the advanced stage of illness at which referrals occur. This aligns with existing evidence indicating late PC referral patterns in kidney disease, often limited to the terminal phase, thereby reducing opportunities for holistic symptom management, psychosocial support, and shared decision-making [16]. 

Dialysis-related decisions further underscore the severity of illness, reflecting thoughtful deliberation regarding treatment futility and burdens. These numbers are consistent with global observations that dialysis withdrawal accounts for 15–25% of deaths among patients receiving maintenance dialysis and is frequently associated with improved alignment with patient preferences [23, 24]. 

The finding that 75.5% of documented deaths occurred in the hospital reflects the high mortality burden and predominantly hospital-centric end-of-life trajectories characteristic of advanced renal disease. This pattern also suggests late referrals in the course of CKD, which may limit opportunities for earlier symptom optimization, advance care planning, and home-based or community-based end-of-life care [14, 16]. 

In low- and middle-income countries’ settings, late palliative referrals are shaped by structural barriers such as limited dialysis capacity, high out-of-pocket costs, workforce shortages, and insufficient primary palliative care training [25–27]. Cultural norms favoring family-led decision-making, clinician discomfort with prognostication, inconsistent documentation, and underdeveloped conservative kidney management pathways further delay timely ACP and GOC discussions, contributing to hospital-centric end-of-life care [28, 29]. 

These findings point to clear policy priorities: development of national KSC guidelines with defined referral criteria and documentation standards; strengthening nephrology workforce capacity through focused communication training and task-sharing; and implementing standardized EMR templates and key performance indicators [30, 31]. Financing mechanisms that support KSC consultations and conservative kidney management are also essential [9]. 

### Strengths and limitations

This study draws strength from its large cohort over six years and comprehensive data capturing referral patterns, interventions, and treatment decisions in advanced kidney disease. It also contributes valuable evidence from a low- and middle-income country context, where Kidney Supportive Care is less studied. The very low frequency of documented ACP discussions likely reflects both late-stage referral patterns and under-documentation within retrospective clinical records.

However, its retrospective, single-center design limits generalizability and depends on the quality of documentation, with limited data on patient-reported outcomes and infrequent recording of advance care planning. Clinician-driven referrals without standardized triggers may also introduce variability. We did not perform multivariable analyses to identify independent predictors of clinical outcomes or mortality. This was due to limitations in data completeness, variable standardization, and the retrospective nature of documentation. As a result, the findings are descriptive and hypothesis-generating, and causal inferences cannot be drawn.

### Future implications

The findings of this study highlight several opportunities to strengthen Kidney Supportive Care integration within nephrology services. Establishing standardized referral criteria and early screening for supportive care needs may help shift referrals to earlier stages of CKD, enabling more proactive symptom management and patient-centered decision-making. Expanding advance care planning into routine nephrology practices, supported by clinician training and structured documentation tools, will be essential for promoting value-based treatment choices and reducing crisis-driven decision-making.

## Conclusion

This review highlights the high clinical complexity and hospital-centric end-of-life course in advanced renal disease, with Palliative Medicine playing a key role in decision-making, symptom control, and goal-of-care transitions. The findings underscore the need to integrate Kidney Supportive Care into routine nephrology practice through structured ACP protocols, routine discussions about prognosis, and time-limited dialysis trials. Variability in documentation indicates the value of standardized referral criteria, uniform EMR templates, and clear pathways to ensure equitable access. Ongoing evaluation and quality-improvement efforts are crucial to advancing coordinated, person-centered kidney care.

## Data Availability

The underlying research materials, including anonymized patient-level data and statistical analysis scripts, are available from the corresponding author upon reasonable request. Due to ethical and privacy considerations, raw clinical data cannot be shared publicly, but de-identified datasets and analytic code will be provided to qualified researchers who meet institutional and regulatory requirements.

## Declarations

### Use of artificial intelligence (AI)

No generative artificial intelligence tools were used in the design, data collection, analysis, or interpretation of this study. AI-assisted tools were used only for language editing and manuscript structuring, with all content reviewed and approved by the authors.
